# Perspective on fibrinolytic therapy in COVID-19: the potential of inhalation therapy against suppressed-fibrinolytic-type DIC

**DOI:** 10.1186/s40560-020-00491-y

**Published:** 2020-09-18

**Authors:** Hidesaku Asakura, Haruhiko Ogawa

**Affiliations:** 1 Department of Hematology, Kanazawa University Hospital, Takaramachi 13-1, Kanazawa, Ishikawa 920-8640 Japan; 2 Department of Environmental and Preventive Medicine, Kanazawa University, Takaramachi 13-1, Kanazawa, Ishikawa 920-8640 Japan

**Keywords:** COVID-19, Thrombosis, Fibrinolytic therapy

## Abstract

A high rate of thrombotic complications, such as pulmonary embolism, has been linked to mortality in COVID-19, and appropriate treatment of thrombosis is important for lifesaving. Although heparin is frequently used to treat thrombotic pathology in COVID-19, pulmonary embolism is still seen in severe cases. Although systemic fibrinolytic therapy is a focus of attention because a thrombotic pathology is the cause of death in severe COVID-19, it should be kept in mind that fibrinolytic therapy might be harmful at advanced stage of COVID-19 where the status of disseminated intravascular coagulation (DIC) has been transmitted from suppressed-fibrinolytic to enhanced-fibrinolytic in disease progression of COVID-19. In this respect, inhalation therapy with fibrinolytic substances might be a safe and promising treatment.

## Fibrinolytic therapy for pulmonary intravascular coagulation in coronavirus disease 2019 (COVID-19)

Some articles suggest several treatment options using fibrinolytic drugs for acute respiratory distress syndrome (ARDS) in severe cases of COVID-19 [1–4]. ARDS and organ dysfunction associated with a cytokine storm have been identified as causes of death in COVID-19 [5]. In addition, a high rate of thrombotic complications, such as pulmonary embolism, has been linked to mortality. Interestingly, despite the presence of systemic hypercoagulation associated with the strong inflammation in severe COVID-19, the site of thrombosis was the lungs in the overwhelming majority of cases. These were not only cases of pulmonary embolism diagnosed by methods such as contrast computed tomography, but included microscopic fibrin thrombosis that was frequently seen at the level of the pulmonary microcirculation in autopsy investigations [6–8]. The pulmonary thrombosis in severe COVID-19 can be described as macroscopic and microscopic thrombosis. Although activation of coagulation is linked to a systemic cytokine storm, since the principal site of thrombus formation is the lungs, it might also be referred to as pulmonary intravascular coagulation [9]. COVID-19 patients with a high D-dimer level are known to have poor clinical outcomes, and this is thought to reflect a direct link between the thrombotic pathology and prognosis [10]. As Whyte et al. noted, exposure of tissue factors (TF) on damaged alveolar endothelial cells and on the surface of leukocytes promotes fibrin deposition. They also mentioned that significantly elevated expression of plasminogen activator inhibitor 1 (PAI-1) by lung epithelial and endothelial cells creates a hypofibrinolytic state [1]. Although heparin is frequently used to remedy the thrombotic pathology in COVID-19, pulmonary embolism is still seen in approximately 20% of severe cases, and there is disagreement regarding the intensity of anticoagulant therapy. Besides heparin, the thrombotic condition might be improved by using fibrinolytic drugs to degrade pre-existing fibrin in the lung [1–4]. The fibrinolytic drug, tissue-type plasminogen activator (tPA), has been systemically administered to treat ARDS in COVID-19 and appeared to be effective in some patients [2–4]. Since tPA has been reported to show an anti-inflammatory effect in addition to a fibrinolytic action, this potential of tPA would be helpful for improving the prognosis of COVID-19 patients [11, 12].

## The state of DIC in the advanced-stage of COVID-19 is apparently different from that of DIC induced by sepsis

However, great caution is required when systemically administering tPA for severe COVID-19 associated with ARDS. According to a report by Tang et al., levels of fibrin/fibrinogen degradation products (FDP) and D-dimer increased significantly, and fibrinogen increased due to an inflammatory response following hospital admission in patients who died [13]. In these patients, further striking increases in FDP (> 120 μg/mL), accompanied by moderate D-dimer elevation (approximately 20 μg/mL), were seen as the patient’s condition worsened. Consequently, a large divergence was seen between FDP and D-dimer levels. In addition, the fibrinogen level rapidly decreased from approximately 4.5 g/L (day 7) to 1.0 g/L (day 10) over a period of just 3 days. The marked increase in FDP, divergence of FDP, and D-dimer levels and marked decrease in the fibrinogen level are findings characteristic of enhanced-fibrinolytic-type DIC and not of DIC associated with sepsis [13, 14]. The type of DIC appears to change from suppressed-fibrinolytic to enhanced-fibrinolytic with disease progression in COVID-19. Plasma tPA levels had been reported to be significantly (*p* < 0.0001) higher in severe cases of COVID-19 (ICU) than in mild cases (Non-ICU). This may indicate that fibrinolysis would be more activated in the advanced phase of COVID-19 [15].

## Adverse effects of systemic thrombolytic therapy for the treatment of COVID-19-related ARDS

The marked decrease in fibrinogen is extremely dangerous if systemic fibrinolytic therapy is administered at the same time, and it gives rise to concerns about fatal bleeding, including cerebral hemorrhage. Particularly in severe cases of COVID-19, the coagulation and fibrinolysis pathology might change greatly over a short period. In previous reports of coagulation and fibrinolysis testing in COVID-19, the overwhelming focus was on D-dimer, followed by PT and APTT. However, periodic testing of fibrinogen and FDP levels is also important. If the results of these tests indicate the presence of enhanced-fibrinolytic-type DIC, systemic fibrinolytic therapy should not be administered.

## Advantageous effects of inhaled thrombolytic therapy beyond systemic therapy

On the other hand, treatment by inhalation of fibrinolysis-related substances, such as tPA and plasminogen, can be administered at any stage of COVID-19 without concerns about bleeding [1, 16]. Administration of fibrinolysis-related substances by inhalation might improve alveolar ventilation by resolving fibrin-containing exudates in the pulmonary alveolar space and dissolving fibrin thrombi at the level of the microcirculation near the alveoli. Inhalation therapy with tPA has gradually been reported to be efficacious for various situations of ARDS or plastic bronchitis [1]. In fact, a phase II clinical trial of tPA inhalation is currently underway (PLATyPuS; alteplase, NCT02315898). Furthermore, randomized, controlled trials of plasminogen inhalation targeting COVID-19 are also ongoing [16].

**Fig. 1:**
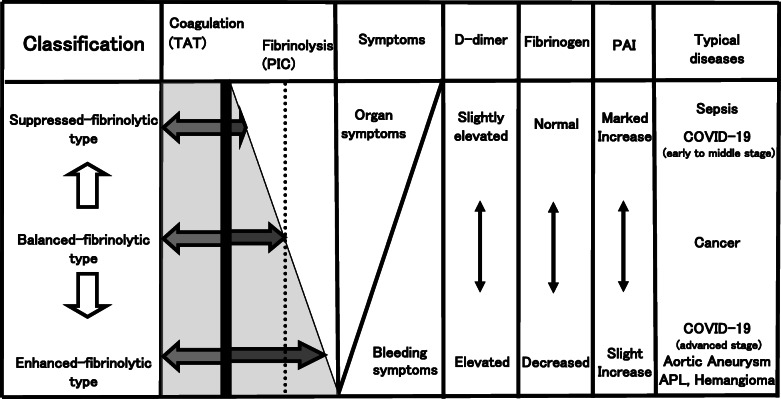
DIC classification. DIC in COVID-19 has previously been discussed exclusively in terms of suppressed-fibrinolytic-type DIC. However, enhanced fibrinolysis results in fatal bleeding, and it therefore requires the planning of anticoagulant therapy that accurately assesses the moment by moment changes in coagulation and fibrinolysis abnormalities. TAT, thrombin-antithrombin complex; PIC, plasmin-α_2_ plasmin inhibitor complex; PAI, plasminogen activator inhibitor; APL, acute promyelocytic leukemia. This figure was previously published by one of the authors (Asakura, 2014) [14] and modified for this manuscript

With systemic fibrinolytic therapy for ARDS in severe COVID-19, there is concern about massive bleeding if the disease stage or coagulation and fibrinolysis pathology is misjudged. Autopsy investigations showed the frequency of both thrombosis and bleeding findings to be the same even if fibrinolytic therapy had not been administered [7, 8]. Taken together with the data for parameters such as FDP, D-dimer, and fibrinogen [13, 14], this indicates that enhanced-fibrinolytic-type DIC is present in advanced stage COVID-19 (Fig. 1).

Although systemic fibrinolytic therapy is a focus of attention because a thrombotic pathology is the cause of death in severe COVID-19, it should be kept in mind that there are cases in which such therapy might be harmful. Indeed, five of 62 patients (8%) with DIC caused by severe meningococcal sepsis had been reported to develop intracerebral hemorrhages after systemic tPA administration [17]. In this respect, inhalation therapy with fibrinolytic substances might be a safe and promising treatment [1, 15]. In fact, inhalation of streptokinase (another fibrinolytic drug) for ARDS demonstrated improvement in the PaO_2_/FiO_2_ ratio without the side effect of bleeding. It is noteworthy that no effect on PT, APTT, or bleeding time has been shown [18]. In the murine study, no side effects of bleeding were reported after inhalation of tPA [19].

Inhalation therapy with fibrinolytic substances should be planned to start at the phase of suppressed-fibrinolytic-type DIC (slightly elevated levels of D-dimer and normal range of fibrinogen) (Fig. 1) or without DIC (normal range of platelet counts) for safety usage. Further studies to evaluate plasma levels of tPA both in inhalation and systemic fibrinolytic therapy would be required to confirm the harmlessness of inhalation therapy.

## Conclusion

Despite the fact that the coagulation/fibrinolytic condition changes in a short period in the DIC of COVID-19, it is of concern that the DIC in COVID-19 is generally understood to be hypercoagulable DIC in many papers. In the absence of appropriate monitoring of the changing state of DIC, systemic fibrinolytic therapy would be considered dangerous.

The clinical efficacy and safety of inhalation of fibrinolytic agents have not been sufficiently investigated, and it is also unclear whether they would be evenly distributed to all alveoli of COVID-19 patients. Moreover, inhalation therapy for patients with COVID-19 may stimulate the production of deleterious aerosols. Even though clinical research is still required, we believe that this new therapy would be good news for COVID-19 patients with ARDS.

## Data Availability

N/A

## Abbreviations

ARDS: Acute respiratory distress syndrome
COVID-19: Coronavirus disease 2019
TF: Tissue factors
PAI-1: Plasminogen activator inhibitor 1
tPA: Tissue-type plasminogen activator
FDP: Fibrin/fibrinogen degradation products
